# Epidemiology and Genetic Characteristics of Carbapenem-Resistant Escherichia coli in Chinese Intensive Care Unit Analyzed by Whole-Genome Sequencing: a Prospective Observational Study

**DOI:** 10.1128/spectrum.04010-22

**Published:** 2023-02-21

**Authors:** Ying Ding, Hemu Zhuang, Junxin Zhou, Lijie Xu, Yi Yang, Jintao He, Min Liang, Shicheng Jia, Xiuliu Guo, Xinhong Han, Haiyang Liu, Linghong Zhang, Yan Jiang, Yunsong Yu

**Affiliations:** a Department of Intensive Care Unit, Sir Run Run Shaw Hospital Qiantang Campus, Zhejiang University School of Medicine, Hangzhou, Zhejiang Province, China; b Department of Infectious Diseases, Sir Run Run Shaw Hospital, Zhejiang University School of Medicine, Hangzhou, Zhejiang Province, China; c Key Laboratory of Microbial Technology and Bioinformatics of Zhejiang Province, Hangzhou, Zhejiang Province, China; d Regional Medical Center for National Institute of Respiratory Diseases, Sir Run Run Shaw Hospital, Zhejiang University School of Medicine, Hangzhou, Zhejiang Province, China; e Department of Pharmacy, Sir Run Run Shaw Hospital Xiasha Campus, Zhejiang University School of Medicine, Hangzhou, Zhejiang Province, China; f Shantou University Medical College, Shantou, Guangdong Province, China; Universität Greifswald

**Keywords:** CREC, carbapenemases, transmission, whole-genome sequencing, disinfectant

## Abstract

This 4-month-long prospective observational study investigated the epidemiological characteristics, genetic composition, transmission pattern, and infection control of carbapenem-resistant Escherichia coli (CREC) colonization in patients at an intensive care unit (ICU) in China. Phenotypic confirmation testing was performed on nonduplicated isolates from patients and their environments. Whole-genome sequencing was performed for all E. coli isolates, followed by multilocus sequence typing (MLST), and antimicrobial resistance genes and single nucleotide polymorphisms (SNPs) were screened. The colonization rates of CREC were 7.29% from the patient specimens and 0.39% from the environmental specimens. Among the 214 E. coli isolates tested, 16 were carbapenem resistant, with the *bla*_NDM-5_ gene identified as the dominant carbapenemase-encoding gene. Among the low-homology sporadic strains isolated in this study, the main sequence type (ST) of carbapenem-sensitive Escherichia coli (CSEC) was ST1193, whereas the majority of CREC isolates belonged to ST1656, followed by ST131. CREC isolates were more sensitive to disinfectants than were the carbapenem-resistant Klebsiella pneumoniae (CRKP) isolates obtained in the same period, which may explain the lower separation rate. Therefore, effective interventions and active screening are beneficial to the prevention and control of CREC.

**IMPORTANCE** CREC represents a public health threat worldwide, and its colonization precedes or occurs simultaneously with infection; once the colonization rate increases, the infection rate rises sharply. In our hospital, the colonization rate of CREC remained low, and almost all of the CREC isolates detected were ICU acquired. Contamination of the surrounding environment by CREC carrier patients shows a very limited spatiotemporal distribution. As the dominant ST of the CSEC isolates found, ST1193 CREC might be considered a strain of notable concern with potential to cause a future outbreak. ST1656 and ST131 also deserve attention, as they comprised the majority of the CREC isolates found, while *bla*_NDM-5_ gene screening should play an important role in medication guidance as the main carbapenem resistance gene identified. The disinfectant chlorhexidine, which is used commonly in the hospital, is effective for CREC rather than CRKP, possibly explaining the lower positivity rate for CREC than for CRKP.

## INTRODUCTION

Carbapenem-resistant Escherichia coli (CREC) infections are an increasingly severe public health problem, imposing a heavy blow to socioeconomic and health care delivery systems, potentially even causing a global health crisis. CREC is prevalent in developed countries, and its widespread transmission tends to occur in contaminated health care settings rather than in the community. The most common mode of transmission is from one patient to another, primarily via the hands (1). Prior to 2001, the Greek Antimicrobial Drug Resistance Surveillance System reported a carbapenem resistance prevalence of <1%, which increased to 30% in hospital wards and 60% in intensive care units (ICUs) within 7 years (2). Studies in the United States demonstrate that for every 1% increase in colonization pressure, the CREC infection odds increase by 15%. In a study conducted in the Chicago, IL, area, 30% of long-term-hospitalized patients were found to be CREC carriers, and the rate was 3.3% for patients in the ICU. Hospitals have become the major sites of CREC infection transmission (3). E. coli is normally found in the human intestinal tract and can cause diseases under certain conditions.

The causes of carbapenem resistance are complex and heterogeneous. A recent CRACKLE-2 study found that 31% of CREC isolates from the United States contain carbapenemases (4). This rate is as high as 90% in the Middle East and Africa. Geographic heterogeneity is a new challenge in CREC control. The mortality rate of patients with CREC infections in the ICU reaches up to 45.5%, and the infection prolongs hospital stays and increases the economic burden on patients (5).

As a worldwide emphasis on CREC research continues, the World Health Organization has identified CREC as a critical priority pathogen for novel drug development. In addition to the ongoing development of new treatments, strengthening antimicrobial stewardship will be vital to ensuring the continued effectiveness of these treatments in the coming years. The epidemiology and wealth of data generated have demonstrated the urgent need for management of CREC; however, the scientific quality of the evidence supporting the management of CREC infection, especially in assessing antibiotic effectiveness in complex patients, remains low due to the lack of strong randomized controlled trials (6).

Given the lack of data on CREC in the ICU in China, the aim of this study was to explore its relevant epidemiological characteristics. Genomic methods were used to understand the distribution of CREC, genetic pattern, genetic relationship to the corresponding CREC, and relationship with CREC transformation. Toward this end, we conducted a prospective observational study in a 28-bed ICU of a tertiary teaching hospital in China. Genomic sequences were recorded, along with epidemiological and medication administration data, to investigate the diversity of CREC in the ICU and the association between CREC infection, the host, and the environment.

## RESULTS

### Study design and CREC collection.

**FIG 1:**
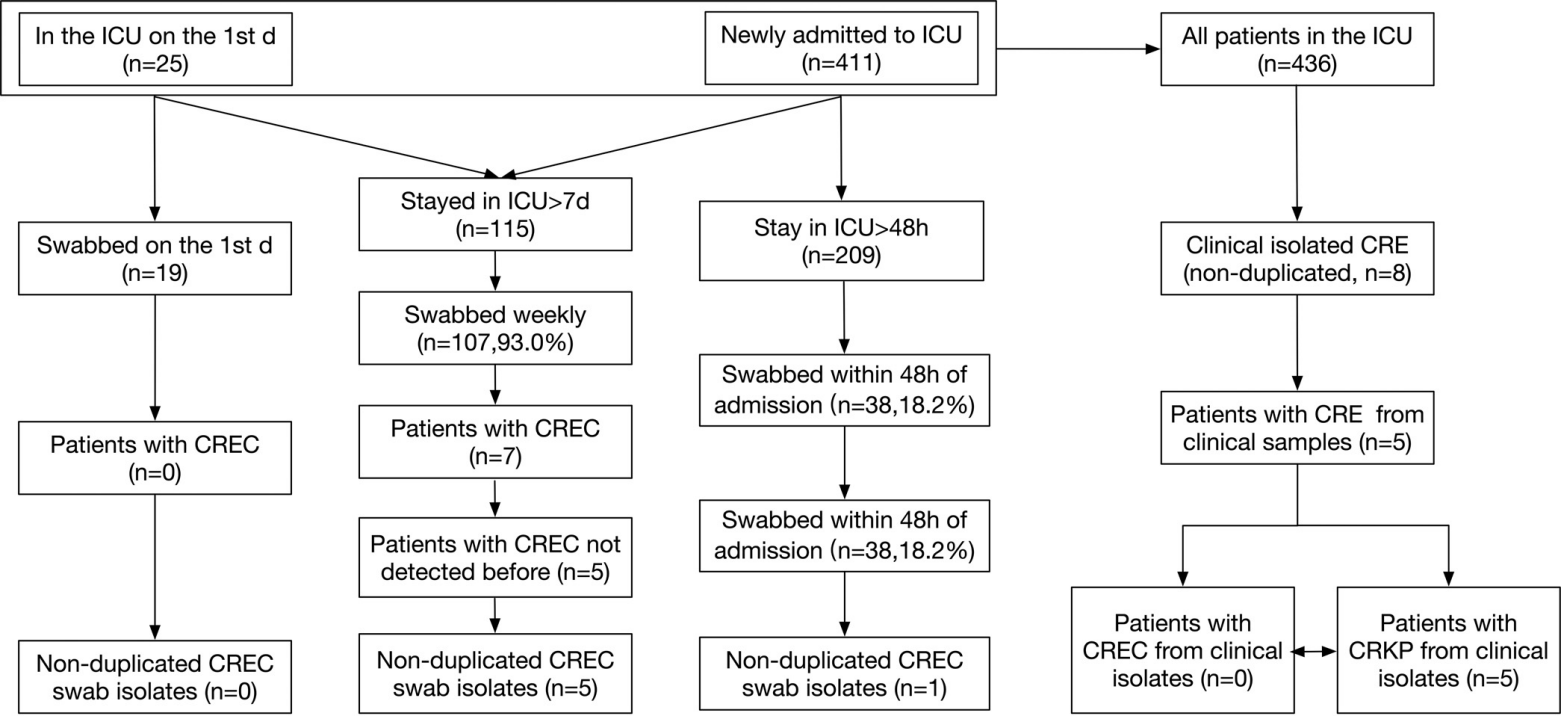
Strain screening method for clinical samples in this study. Strains isolated from oropharyngeal swabs, rectal swabs, nasogastric tubes, nasogastric tubes, tracheal intubation tubes, and tracheotomy tube swabs from the same patient were defined as nonduplicate samples, while strains isolated from the same swab type from the same patient in the same week were defined as duplicate samples. Patients who were already in the intensive care unit (ICU) on the first day of the study without detection of carbapenem-resistant Escherichia coli (CREC) or who were newly admitted to the ICU with negative results of CREC screening within 48 h were defined as patients without CREC.

The study duration was 4 months, with 17 weekly sample collections from 436 patients. On day 1 (1 April 2021), 25 patients were admitted to the ICU (Fig. 1). We collected samples, including rectal swabs and oropharyngeal swabs, from 19 of the 25 patients, none of which contained CREC. Of the 411 new ICU admissions during the study period, 209 were admitted to the ICU for longer than 48 h (Fig. 1). Samples (i.e., oropharyngeal and rectal swabs) were collected from 38 of these 209 patients (18.2%) within 48 h of admission. Only 1 of these 38 patients tested positive for CREC. Patient 4 (P4) was admitted to a local hospital with a suspected trauma infection. We considered this patient’s CREC infection to be non-ICU acquired, as CREC was detected on the rectal and gastric tube swabs on the second day of his transfer to the ICU. The carriage rate of CREC at admission was 2.6% (1/38).

Five patients were believed to have ICU-acquired CREC. The first patient (P2) had a negative rectal swab on day 5 of ICU admission, followed by two positive swabs thereafter. The second patient (P3) had negative rectal swabs on days 2 and 9 after ICU admission and a positive rectal swab on day 16. The third patient (P6) was identified to have carbapenem-sensitive E. coli (CSEC) in a rectal swab on day 9 after admission, and CREC was detected in a gastric tube sample taken on day 16 of his ICU stay. The fourth patient (P7) had negative rectal, tracheal intubation, and nasogastric tube swabs on day 4 after ICU admission, and CREC was identified in a rectal swab on day 11 after ICU admission. The last patient (P8) was identified to have CSEC in the first two rectal swabs after ICU admission, and CREC was detected in the rectal swab on day 17.

During the study period, eight nonreplicate clinical isolated carbapenem-resistant *Enterobacteriaceae* (CRE) were found in five different patients: five with carbapenem-resistant Klebsiella pneumoniae (CRKP) and none with CREC (Fig. 1).

### Screening for CREC in environmental samples.

We collected a total of 5,824 environmental specimens, 23 of which were positive for E. coli, thus leading to a detection rate of 0.39%. The 23 E. coli-positive environmental samples were from 18 patients, distributed in 13 of 28 beds. E. coli was detected in several environmental samples, including the overflow wall, inner surface of the downspout, nebulizer, bedside table, bed rails, ventilator, micropump, bed linen, hanging cabinet, faucet surface, stethoscope, bed regulator, and locker. Among these 23 samples in which E. coli was detected, 3 were CREC-positive environmental samples from the nebulizer (P4, DY315), bedside cabinet (P4, DY316), and faucet surface (P9, DY317). One patient (P4) had CREC of the same strain, confirmed by genotype sequencing, detected in his rectal swab, nasointestinal tube, and surroundings (nebulizer and bedside table). The other patient (P9) did not carry CREC, but CREC was detected on the faucet surface of his room. Genome sequencing revealed that this strain was homologous (difference of 22 SNPs) to that found in the rectal swab sampled from a patient (P5) in the adjacent room during the same period. The weekly sampling during this patient’s (P4) hospitalization identified two nonduplicate environmental samples of CREC, from the nebulizer (P4, DY315) and bedside table (P4, DY316), only for the duration of 1 week.

### Molecular characteristics of E. coli.

**FIG 2:**
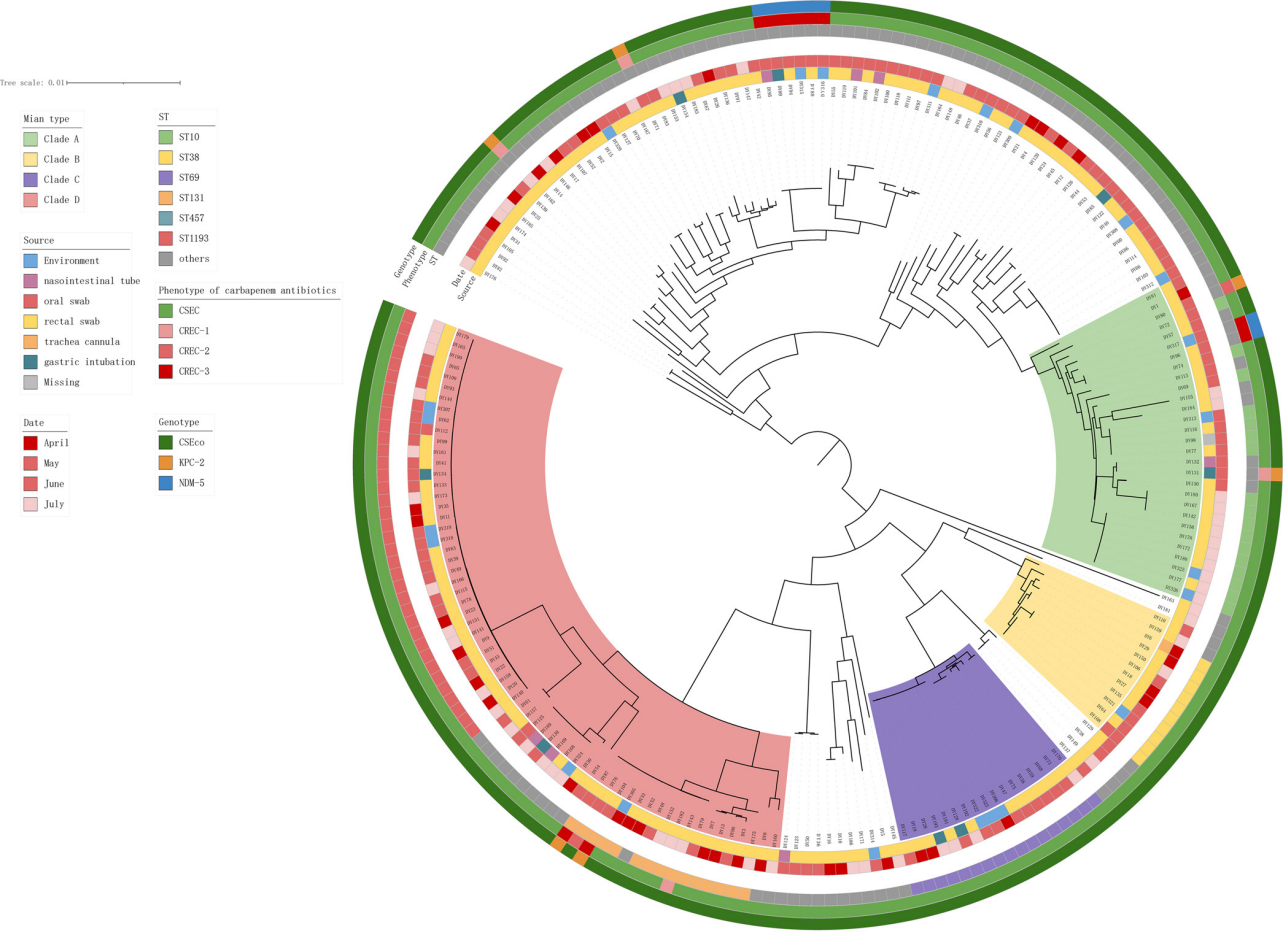
Phylogenetic tree constructed by the maximum likelihood method reflecting the genetic relationships among all 214 E. coli strains. From inside to outside, the main branches (divided into four branches), sample source, collection time, possible infection site, sequence type (ST), phenotype, and genotype for all E. coli strains are successively listed in each layer. In the phenotype circle, we defined E. coli resistance to one, two, and three kinds of carbapenem drugs as CREC-1, CREC-2, and CREC-3, respectively.

A total of 214 E. coli strains were isolated, of which 191 were isolated from 99 different patients and 23 were isolated from the surrounding environment in the ICU ward. Multilocus sequence typing (MLST) divided the 214 E. coli isolates into four clades: clade A, containing 29 strains, mainly sequence type (ST) 10; clade B, containing 12 strains, mainly ST38; clade C, containing 17 strains, mainly ST69; and clade D, containing 64 strains, mainly ST131 and ST1193 (Fig. 2). Among the strains isolated in this study, ST1193 (*n* = 36 [16.82%]) was the most prevalent, and all of them clustered in clade D. This was followed by ST10 (*n* = 18 [8.41%]), ST69 (*n* = 17 [7.94%]), and ST131 (*n* = 15 [7.01%]). In clade A, we found four strains of CREC, including two with the *bla*_KPC-2_ gene, both with *bla*_NDM-5_, and none with ST10. In clade D, we found four strains of CREC, including two with the *bla*_KPC-2_ genes and two without carbapenem resistance genes, all belonging to ST131. The remaining strains exhibiting carbapenem resistance did not belong to the above four clades, whereas previous related studies have shown that most CREC strains belong to ST167, followed by ST410 and ST131, and most carry various *bla*_NDM-5_ genes (7). ST1193 was the common ST in the E. coli detected in this study (46 strains [21.5%]). In contrast, most of these carbapenem-resistant strains did not belong to the aforementioned STs.

### CREC characterization.

**FIG 3:**
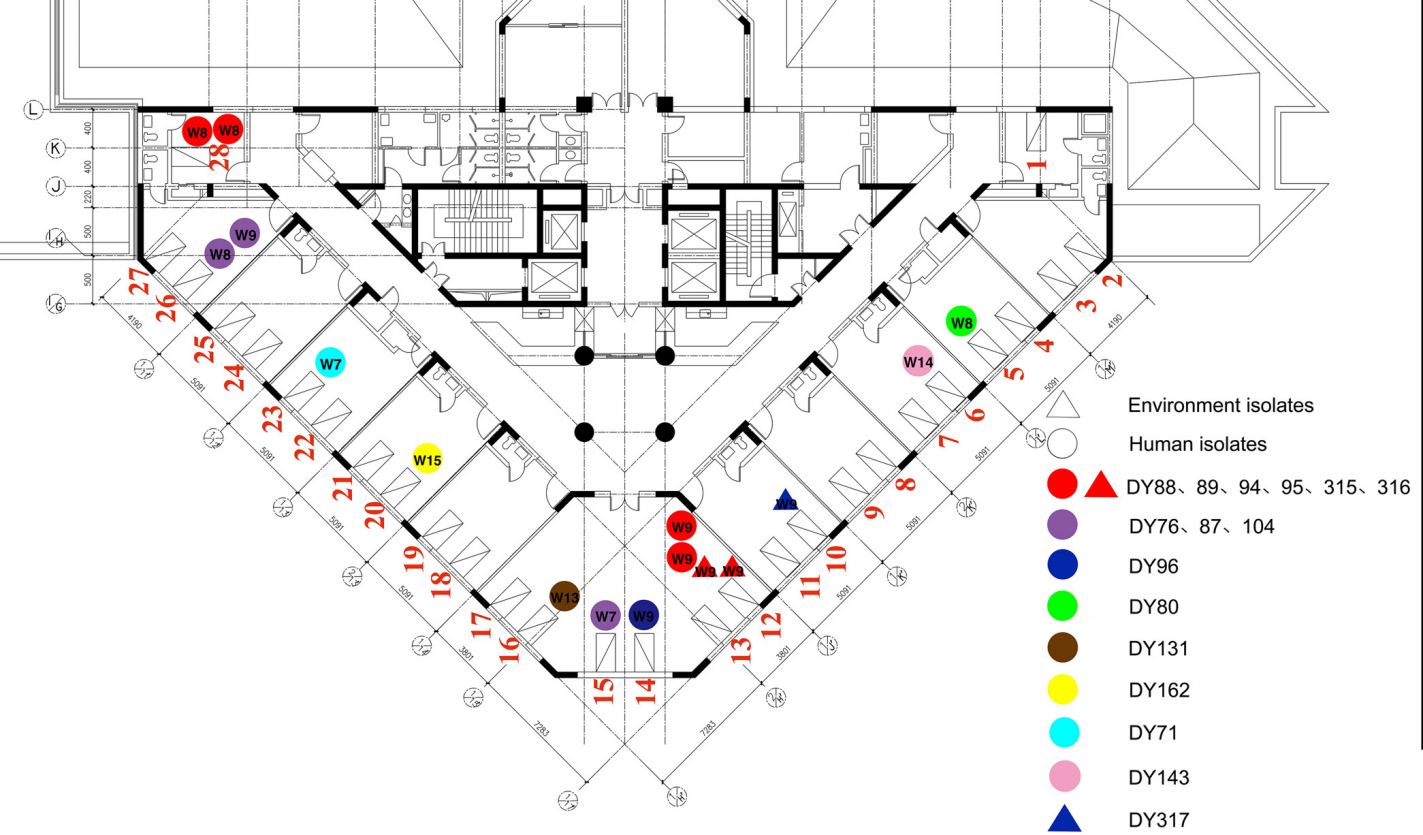
ICU floor sketch map and overall distribution of CREC strains. The ICU on the fourth floor of the hospital has a total of 28 beds, including two single rooms (no. 1 and 28), one six-bed room (no. 12, 13, 14, 15, 16, and 17), and 10 two-bed rooms (remaining rooms). Strains from the same bed can be a superposition of positive results collected at different times. Clinical samples are represented by circles, and environmental samples are represented by triangles. The number of the week when the sample was collected is shown on the circle or triangle. Different colors represent CREC strains with different single nucleotide polymorphism (SNP) typing.

Sixteen carbapenem-resistant E. coli strains, from patients occupying 11 of the 28 beds, were detected in this study (Fig. 3). Two carbapenemases were detected among them: KPC-2 and NDM-5. NDM-5 was the most common (50.00% [8/16]), followed by KPC-2 (37.50% [6/16]), whereas no carbapenemase was detected in two isolates (12.50% [2/16]). Moreover, 15 other β-lactamases were detected (Table 1). According to the antimicrobial susceptibility test results, the rate of resistance to meropenem was 64.70% (11/17), with a MIC range of 0.03 to >256 mg/L. The rate of resistance to imipenem was 64.70% (11/17), with a MIC range of 0.25 to >256 mg/L. The rate of resistance to ertapenem was 100%. The MIC values and ranges for the nine different antibiotics were tested (Table 1).

**TABLE 1:** MIC values of all CREC strains for three carbapenems and other antibiotics, their STs, the genotypes of carbapenemases, the Inc type of carbapenem-carrying plasmid, and other β-lactamase genes^*a*^

Patient ID	Isolated strain	MLST	Carbapenemase	Inc type of carbapenem-carrying plasmid	Other β-lactamase(s)	MIC (mg/L)					
						MEM	IPM	ETP	COL	TGC	CZA
1	DY71	ST101	KPC-2	IncFII	CTX-M-14, TEM-1, EC-18	1	2	4	0.5	0.25	0.5
2	DY76	ST131			CTX-M-27, EC-5	4	2	32	1	0.125	0.5
3	DY80	ST3337	KPC-2	IncN	CTX-M-3, TEM-1, EC	1	4	4	1	0.5	0.25
2	DY87	ST131	KPC-2	IncN	CTX-M-27, CTX-M-3, TEM-1, EC-5	>256	>256	>256	1	0.25	1
4	DY88	ST1656	NDM-5	IncHI2	OXA-10, TEM-1, EC-18	128	256	128	0.5	0.25	>128
4	DY89	ST1656	NDM-5	IncHI2	OXA-10, TEM-1, EC-18	8	16	32	1	0.125	>128
4	DY94	ST1656	NDM-5	IncHI2	OXA-10, TEM-1, EC-18	16	16	32	0.5	0.25	>128
4	DY95	ST1656	NDM-5	IncHI2	OXA-10, TEM-1, EC-18	4	8	16	0.5	0.25	>128
5	DY96	ST6913	NDM-5	IncFIB/IncFIC	EC	8	8	32	1	0.25	>128
2	DY104	ST131	KPC-2	IncN	CTX-M-27, CTX-M-3, TEM-1, EC-5	>256	>256	>256	0.5	0.125	1
6	DY131	ST744	KPC-2	IncN	CTX-M-14, CTX-M-3, TEM-1, EC	2	2	4	0.5	0.125	0.5
7	DY143	ST131			CTX-M-14, CTX-M-15, OXA-1, TEM-1, EC-5	2	1	16	0.5	0.125	0.25
8	DY162	ST410	KPC-2	IncN	CTX-M-3, TEM-1, EC-15	1	1	2	1	0.125	<0.0625
4	DY315	ST1656	NDM-5	IncHI2	OXA-10, TEM-1, EC-18	8	4	4	0.5	0.25	>128
4	DY316	ST1656	NDM-5	IncHI2	OXA-10, TEM-1, EC-18	16	16	32	1	0.125	>128
9	DY317	ST6913	NDM-5	IncFIB/IncFIC	EC	16	16	16	1	0.5	>128

a CREC, carbapenem-resistant E. coli; ST, sequence type; ID, identifier; MLST, multilocus sequence type; MEM, meropenem; IMP, imipenem; ETP, ertapenem; COL, colistin or polymyxin E; TGC, tigecycline; CZA, ceftazidime-avibactam.

MLST analysis classified the 16 carbapenem-resistant E. coli strains into seven different STs (Table 1). ST1656 was predominant (37.50% [6/16]), followed by ST131 (25.00% [4/16]) and ST6913 (12.50% [2/16]).

**FIG 4:**
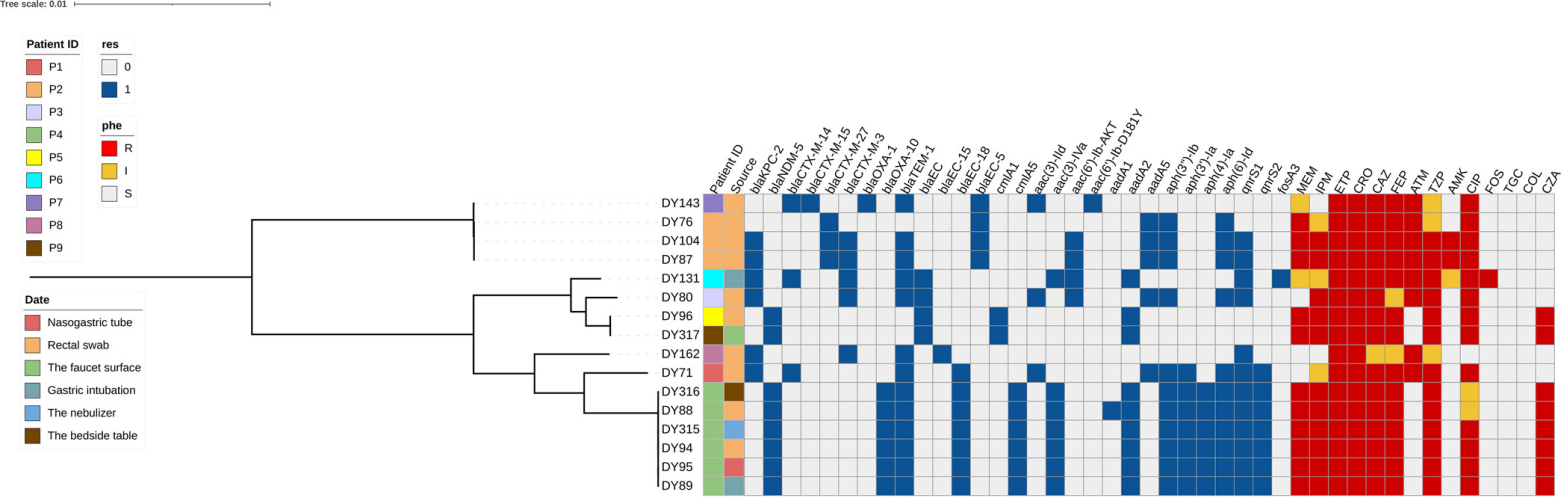
Resistance genotypes and drug-sensitive phenotypes of 16 nonreplicate samples of CREC detected in 9 patients categorized into four classes of antibiotics (β-lactams, aminoglycosides, quinolones, and fosfomycin) that are mainly used as clinical anti-infection strategies.

The presence of known resistance genes was screened in 16 carbapenem-resistant isolates. In total, 29 acquired antibiotic resistance genes were identified, conferring resistance to five classes of antibiotics. These included genes encoding extended-spectrum β-lactamases (*bla*_CTX-M-14_, *bla*_CTX-M-15_, *bla*_CTX-M-27_, *bla*_CTX-M-3_, *bla*_OXA-1_, and *bla*_OXA-10_), genes conferring resistance to aminoglycoside antibiotics [*aac(3)-IId*, *aac(3)-Iva*, *aac(6′)-Ib-AKT*, *aac(6′)-Ib-D181Y*, *aadA1*, *aadA2*, *aadA5*, *aph(3″)-Ib*, *aph(3′)-Ia*, *aph(4)-Ia*, and *aph(6)-Id*], genes conferring resistance to quinolone antimicrobials (*qnrS1*, *qnrS2*, *oqxA10*, and *oqxB17*), genes conferring resistance to fosfomycin antibiotics (*fosA3*), and genes conferring resistance to chloramphenicol antibiotics (*cmlA1* and *cmlA5*) (Fig. 4).

Isolates DY96 and DY317, from different patients, were identified to be ST10 and had a close evolutionary relationship, based on a short distance in the phylogenetic tree. Single nucleotide polymorphism (SNP) analysis revealed that they differed by 22 SNPs, indicating that they were of the same origin and that transmission between patient and environment had likely occurred.

A total of five different plasmids carrying carbapenem resistance genes were obtained from 16 strains of CREC isolated from nine patients, three carrying *bla*_KPC-2_ and two carrying *bla*_NDM-5_. The *bla*_KPC-2_-carrying plasmid of DY71 (isolated from patient 1) belonged to the IncFII replicon and was identical to the *bla*_KPC-2_-harboring plasmid pC110-KPC (GenBank accession number CP047692.1) isolated from Serratia marcescens strain C110 (see Fig. S1 in the supplemental material). The *bla*_KPC-2_ gene in DY71 was flanked by IS*Kpn27* and IS*Kpn6* (Fig. S1). Strains DY80, DY87, DY104, and DY131 (isolated from patients 2, 3, and 6) had the same *bla*_KPC-2_-carrying IncN-type plasmid that was highly similar to pCRKP-1-KPC (GenBank accession number KX928750.1) carried by a K. pneumoniae strain (Fig. S2). These *bla*_KPC-2_ genes were flanked by IS*26*, IS*Kpn27*, and IS*Kpn19* (Fig. S2). It is worth noting that the *bla*_KPC-2_-harboring plasmid of the DY162 strain (isolated from patient 8) is 36,597 bp and its nucleotide sequence is the same as a part of the pCRKP-1-KPC plasmid mentioned above (Fig. S2). Unfortunately, the *bla*_NDM-5_-carrying plasmid in this study could not be assembled completely. Based on the obtained long contig, we found that strains DY88, DY89, DY94, DY95, DY315, and DY316 (isolated from patient 4) had the same *bla*_NDM-5_-carrying plasmid that was highly similar to the reported pEC6622-1 isolated from E. coli (GenBank accession number CP096588). This plasmid belonged to the IncHI2 replicon, and the *bla*_NDM-5_ gene was flanked by IS*3000*, IS*Kpn26*, and IS*26* (Fig. S3). We also found that strains DY96 and DY317 (isolated from patients 5 and 9) had the same *bla*_NDM-5_-carrying plasmid, which was highly similar to the reported plasmid pYUXYEH3783-NDM (GenBank accession number CP110998) (Fig. S4). This plasmid belongs to the IncFIB/IncFIC replicons, and *bla*_NDM-5_ is surrounded by IS*26*, IS*Kpn26*, and IS*Aba125*.

### Disinfectant sensitivity of the strains.

To explore why CREC was isolated at a much lower frequency than CRKP, we evaluated the resistance of these strains to disinfectants. We also identified 16 CRKP strains that belong to multiple STs corresponding to the same period of collection as the 16 CREC strains (Table 2). The chlorhexidine MICs for 16 CREC strains ranged from 0.5 to 1 mg/L, while the corresponding MIC range for 16 CRKP strains was 2 to 16 mg/L. The MIC_90_ for CRKP was eight times higher than that for CREC. However, the MICs of *p*-chlorophenol and calcium hypochlorite for the 16 CREC strains and 16 CRKP strains presented high-level resistance (MIC>256 mg/L) with no significant difference (Table 3).

**TABLE 2:** Information on 16 CREC and CRKP strains^*a*^

Strain date (mo.day)	CREC					CRKP				
	Patient ID	Isolate no.	Bed no.	Sample type	ST	Patient ID	Isolate no.	Bed no.	Sample type	ST
5.18	1	DY71	23	Rectal	101	10	kp1239	8	Rectal	15
5.18	2	DY76	15	Rectal	131	2	kp1240	15	Rectal	43
5.25	3	DY80	5	Rectal	3337	3	kp1282	5	Rectal	43
5.25	2	DY87	26	Rectal	131	2	kp1306	26	Rectal	43
5.25	4	DY88	28	Rectal	1656	11	kp1283	21	Rectal	11
5.25	4	DY89	28	Gastric intubation	1656	12	kp1292	11	Gastric intubation	43
6.01	4	DY94	12	Rectal	1656	13	kp1326	19	Rectal	15
6.01	4	DY95	12	Nasogastric tube	1656	14	kp1319	20	Nasogastric tube	11
6.01	5	DY96	14	Rectal	6913	15	kp1338	4	Rectal	11
6.01	2	DY104	26	Rectal	131	2	kp1328	26	Rectal	43
6.29	6	DY131	16	Gastric intubation	744	6	kp1442	16	Gastric intubation	43
7.06	7	DY143	6	Rectal	131	15	kp1454	23	Rectal	11
7.13	8	DY162	20	Rectal	410	8	kp1479	20	Rectal	483
6.01	4	DY315	12	Nebulizer	1656	16	kp1315	18	Nebulizer	43
6.01	4	DY316	12	Bedside cabinet	1656	17	kp1332	24	Bedside cabinet	15
6.01	9	DY317	10	Faucet surface	6913	18	kp1342	8	Faucet surface	43

a Sampling date, isolate number, bed number, sample type, and ST for 16 CREC and carbapenem-resistant Klebsiella pneumoniae (CRKP) strains collected in the same period.

**TABLE 3:** Susceptibility test results of clinically isolated CREC and CRKP isolates for three disinfectants

Organism	MIC of disinfectant (mg/L)								
	Chlorhexidine			*p*-Chlorophenol			Calcium hypochlorite		
	Range	50%	90%	Range	50%	90%	Range	50%	90%
CREC	0.5–1	0.5	1	256~512	256	512	>256	>256	>256
CRKP	2–16	4	8	256	256	256	>256	>256	>256
ATCC 25922	1			512			>256		
ATCC 700603	16			256			>256		

## DISCUSSION

The emergence of multidrug-resistant Gram-negative bacteria is becoming increasingly serious, causing approximately 700,000 deaths yearly worldwide. This number is expected to reach 10 million by 2050 if no effective solution is found (8). CREC infections are associated with increased mortality, prolonged hospitalization (9, 10), and increased treatment costs compared to CSEC infections (11). An economic prediction model suggested that the increased socioeconomic cost per CREC-infected person ranges from $50,000 to $80,000 (12). First used in the 1980s, carbapenems have become the most effective antibacterial drugs for treating multidrug-resistant Gram-negative bacteria, owing to their advantages of broad-spectrum activity, strong antibacterial effect, stable effect on β-lactamase and cephalosporinase, and low toxicity. Carbapenems have shown good efficacy in the treatment of severe multidrug-resistant bacteria infections (3, 13). However, with extensive clinical use, the detection rate of CREC has gradually increased (14). Asymptomatic CREC colonization precedes or occurs concurrently with infection and is a risk factor for CREC infection, constituting a CREC reservoir (15).

Data from the China Network for Bacterial Resistance Surveillance (CHINET [https://www.chinets.com/Data/AntibioticDrugFast]) showed that the resistance of E. coli to carbapenem antibiotics was relatively stable in China, with rates of 1.1% and 1.4% for imipenem and meropenem, respectively, in 2005, compared with 2.0% and 2.1%, respectively, in 2019. The China Drug Resistance Surveillance Network (http://www.carss.cn/) indicated that the national average rate of resistance of E. coli to carbapenem antibiotics in 2019 was 1.5%, the same as in 2017, with the highest rate in Henan Province at 2.9%, and the overall resistance rate had remained low.

In this study, CREC was detected in only one patient sample taken within 48 h following ICU admission. This patient was admitted to the orthopedic department of a local hospital. Postoperative wound infection was suspected, and carbapenem drugs were administered before ICU admission. He was later transferred to the ICU for continued treatment. Thus, the CREC detected was considered to be hospital acquired. In addition, ICU-acquired CREC from five patients also were hospital-acquired CREC. We can infer that almost all of the CREC cases identified in this study were hospital acquired, and community-acquired CREC is still very rare. Tran et al. (16) also found that the colonization of CREC among patients was mainly hospital acquired, and the rate of CRE colonization increased with the length of ICU stay in a study of 12 hospitals in Vietnam. They also proposed that colonization is a prerequisite for CRE infection.

CREC was detected in the faucet surface sample related to patient P9, the genotype of which was considered similar to that found in a patient (P5) whose rectal swab was taken in the adjacent room during the same period. Since there was no hand-washing sink in the large six-bed ward next door to the patient (P9), it is highly likely that the CREC colonization on the faucet surface was caused by the health care worker or nursing staff managing the patient (P5) in the large ward coming to the hand-washing sink in the next patient’s room (P9). Therefore, we suggest setting up more single isolation rooms, if the hospital’s economic conditions allow, and reducing CREC contamination via the circulation of health care workers by hand hygiene management. Furthermore, because of the low rate of detection of CREC in environmental specimens, we can infer that environmental CREC colonization is limited and that the environmental contamination by CREC carrier patients is highly limited with respect to both the temporal and spatial distribution. This is inextricably linked to our strict hand disinfection management, diligent disinfection of the surrounding environment, and the timely transfer of colonized patients to a single room in our hospital.

Regarding the STs of the strains collected, we found that ST1656 and ST131 were predominant. In NDM-producing E. coli outbreaks globally, ST101, ST405, ST410, ST648, ST156, ST167, and ST131 are the most common clones. ST131 is predominant in India, whereas ST167 is predominant in China. Furthermore, we found that ST410 carrying the *bla*_NDM-5_ gene appears to be of greater concern because it is widely disseminated in China and is known to cause infections throughout the country (17). Among the 16 carbapenem-resistant E. coli strains, 2 lacked carbapenemases. It has been previously reported that the mechanisms of carbapenem resistance are closely related to the production of carbapenemases (acquisition of carbapenemase genes), loss of pore proteins in combination with extended-spectrum β-lactamases (ESBLs), and overexpression of efflux pumps. In our study, the two CREC strains lacking carbapenemases might utilize the latter mechanisms, which remains to be investigated (7).

Regarding the MLST results, the genetic analysis for all strains showed low homology among the isolates. The last decade has witnessed a pandemic of ST1193, a new multidrug-resistant clonal group of E. coli. ST1193 has been demonstrated to produce ESBL and is resistant to fluoroquinolones (100%), trimethoprim-sulfamethoxazole, and tetracycline (18–20). Epidemiological studies across developing and developed countries in Asia, Europe, and America recently suggested that ST1193 has become one of the dominant STs of uropathogenic antimicrobial-resistant E. coli, which induces community onset urinary tract infections in children and adults (20–23). In China’s mainland, ST1193 was first reported in a molecular epidemiology study of 590 nonduplicate E. coli isolates from 30 county hospitals in 2015, suggesting that ST1193 is the second most abundant ST among fluoroquinolone-resistant E. coli strains (24). Several studies indicate the possibility of the bidirectional interspecies transmission of E. coli ST1193 among humans, wildlife (such as Australian silver gulls), domestic animals (25), and companion animals (26, 27), which might be one of the causes of the global pandemic of ST1193. Therefore, there is an urgent requirement for increased surveillance to control the rapid worldwide spread of ST1193, which was also the dominant ST identified in this study. In our study, the *bla*_NDM-5_-carrying plasmids of DY88, DY315, and DY316 that had the same genetic structure were able to conjugate into E. coli J53. Therefore, once the *bla*_NDM-5_-carrying conjugative plasmid is transferred to carbapenem-sensitive E. coli clone ST1193, the epidemic of ST1193 CREC will pose a great threat to public health indeed.

We performed long-read Nanopore sequencing to obtain the complete chromosome and plasmid sequences, which facilitated the analysis of genetic structure and the important mobile elements surrounding resistance genes. In particular, the genes encoding NDM-5 and KPC-2 carbapenemases have attracted attention based on the complete sequence. Three *bla*_KPC-2_-carrying and two *bla*_NDM-5_-carrying plasmids have been further analyzed. The genetic environments, such as transposon or insertion sequence (IS) surrounding the carbapenemase gene, have been clarified, broadening our understanding of the transfer mechanism around these important antimicrobial resistance genes.

Finally, we found that the CREC was more easily sterilized by commonly used disinfectants. In our study, we chose chlorhexidine, *p*-chlorophenol, and calcium hypochlorite for investigation. Our results showed that the MIC_90_ of chlorhexidine for CRKP was 8 mg/L, while that for CREC was 1 mg/L. In addition, we found that calcium hypochlorite and *p*-chlorophenol had higher MICs for CREC and CRKP. Thus, they are not recommended for use as common disinfectants in clinical practice. Bacteria can acquire disinfectant resistance by expressing relevant resistance genes. Most disinfectant resistance genes are usually carried by the chromosome encoding the bacterial efflux pump system (28). Chen et al. (29) showed that CRE strains collected from patients (including CREC and CRKP) displayed various degrees of resistance to commonly used disinfectants. Continuous monitoring of the decreasing susceptibility of multidrug-resistant bacteria to disinfectants helps to effectively control and prevent the spread of superresistant bacteria. Our previous study revealed that the detected CRKP carried disinfectant resistance genes and virulence clusters and recommended the appropriate use of disinfectants to prevent the development of resistance (30).

Based on these findings, we demonstrate the CREC colonization rate and the relevant genomic information of CREC in Chinese ICUs, including its major multilocus sequence analysis, drug resistance genes, nucleotide polymorphisms, and other important information, which provide a basis for further research. We have also offered recommendations for how physicians and other health care workers can effectively reduce CREC colonization in the ICU and prevent its transmission. Combining this study with previous studies related to CRKP, we found that the transmission of CREC is relatively limited, whereas that of CRKP is widespread (31–33). And we explored the reason why CREC was isolated at a much lower frequency than CRKP.

However, this study is limited by being a single-center observational study. In addition, the sample size was quite small, and the rate of acquisition of samples from relevant patients within 48 h of admission was low, meaning that a few patients carrying CREC were likely missed, thereby reducing the evidence-based strength of the study results.

### Conclusion.

In conclusion, the rate of detection of CREC was low in both patients and the environment in this study. Almost all of the CREC strains detected were ICU acquired. Contamination of the surrounding environment by CREC-carrying patients is limited both temporally and spatially. Although all strains showed low and sporadic homology, ST1193 was the primary strain of CSEC detected, indicating that this might be the next potential outbreak strain that requires attention. The next most common strains were ST10 and ST69. ST1656 and ST131 also deserve attention, as they comprise the majority of CREC cases. The *bla*_NDM-5_ gene is relevant to empirical antibiotic use as the main carbapenem resistance gene. The difference in clinical transmission between CREC and CRKP is likely due to their different susceptibilities to disinfectants. The results of the susceptibility testing of disinfectants showed that CREC was more susceptible than CRKP. Therefore, effective interventions and active screening are beneficial to the prevention and control of CREC.

## MATERIALS AND METHODS

### Strains.

This prospective study aimed to explore the colonization, transmission, and distribution of CREC in a tertiary teaching hospital in China. The study period was from 1 April 2021 to 31 July 2021, and screening was performed every Tuesday morning. Screening included 6 sites for each patient and 19 environmental sites around the patient. The study was conducted in the 28-bed ICU of Sir Run Run Shaw Hospital, Zhejiang University School of Medicine. We collected weekly clinical samples such as oropharyngeal, rectal, tracheal intubation, gastric intubation, tracheotomy tube, and nasointestinal tube swabs from all of the patients admitted to the ICU during the study period. Weekly environmental samples were taken from each bed and its surrounding structures, including bed rails, button panels of ventilators, cardiac monitors, microinjection pumps, switch buttons, nebulizers, stethoscopes, hanging towers, bed regulators, bedside tables, computer mice and keyboards, ring hooks of infusion racks, treatment carts, and lockers. We also obtained weekly samples from all ward sinks, including the inside of the downspout, faucet surface, sink countertop, inside surface of the overflow, and the water in the plumbing system.

This study was reviewed and approved by the Ethics Committee of Sir Run Run Shaw Hospital, Zhejiang University School of Medicine (no. 20201217-33).

### Strain isolation, strain identification, and drug and disinfectant resistance assays.

Sampling personnel used Copan swabs to obtain samples from the patients, bed units, and sinks. After sampling, the swabs were placed in tryptic soy broth (TSB) and incubated overnight. Twenty microliters of the bacterial solution was then drawn and applied to CHROMagar E. coli screening plates (CHROMagar, Paris, France) for an overnight culture at 37°C. On the second day, a single colony was picked from each plate and inoculated onto a Mueller-Hinton (MH) agar plate. The divisions were marked, and the plates were cultured overnight at 37°C. On the third day, a single colony from each plate was chosen for matrix-assisted laser desorption ionization–time of flight (MALDI-TOF) mass spectrometry (bioMérieux, Marcy-l’Etoile, France) to confirm that the colonies were E. coli strains. The MICs of some drugs, including meropenem, imipenem, ertapenem, ceftriaxone, ceftazidime, cefepime, aztreonam, piperacillin-tazobactam, amikacin, ciprofloxacin, and fosfomycin, were measured using the agar dilution method, while those of ceftazidime-avibactam, colistin, and tigecycline were measured using the broth microdilution method. Both of these methods are recommended by the American Clinical and Laboratory Standards Institute (CLSI) guidelines (2020 edition) (34). All drug sensitivity breakpoints were referenced to the CLSI 2020 implementation criteria except for tigecycline, which was determined according to the FDA 2019 guideline. Furthermore, we applied the broth microdilution method to obtain the MICs of the three frequently used disinfectants in the clinic, including chlorhexidine, *p*-chlorophenol, and calcium hypochlorite, against the isolated CRKP and CREC strains. The quality control strains were E. coli ATCC 25922 and K. pneumoniae ATCC 700603.

### Whole-genome sequencing and analysis.

Genomic DNA was extracted using a Qiagen minikit (Qiagen, Hilden, Germany) and sequenced using the Illumina HiSeq (Illumina, San Diego, CA) and Oxford Nanopore MinION (Nanopore, Oxford, UK) platforms. Illumina read assembly was performed to obtain draft genomes using the shovill pipeline (version 4.4.5 [https://github.com/tseemann/shovill]). The raw data from Nanopore MinION and Illumina sequencing were assembled using Unicycler pipeline to obtain complete genomes under default parameters (35). The assembled genomes were annotated using Prokka (36) and BacAnt (37). A plasmid map was constructed by importing the gbk file into BRIG (version 0.95). The MLST used in the survey was the Achtman scheme, which was performed using mlst (https://github.com/tseemann/mlst) with the PubMLST data set (38). Resistance genes and plasmids were identified using ABRicate v0.8.13 (https://github.com/tseemann/abricate) with the NCBI AMRFinderPlus (39) and PlasmidFinder (40) databases.

### Homology analysis.

The core genome alignment file was generated using the panaroo pipeline (version 1.2.7) in strict mode (41). The maximum likelihood tree was generated using the iqtree pipeline (version 2.1.2) (42). The snippy pipeline (version 4.4.5 [https://github.com/tseemann/snippy]) was used to calculate the SNPs of the isolates. An SNP distance of ≤22 bp to the nearest neighbor was set as the criterion for indicating that two strains were homologous (43).

### Data availability.

The draft genomes of the isolates in this collection have been deposited in the NCBI database under BioProject accession number PRJNA911045.
