# Supplementary material for: Epidemiology and Genetic Characteristics of Carbapenem-Resistant Escherichia coli in Chinese Intensive Care Unit Analyzed by Whole-Genome Sequencing: a Prospective Observational Study
Source: Microbiol Spectr. 2023 Feb 21;11(2):e04010-22. doi: 10.1128/spectrum.04010-22 (PMC10100791; doi:10.1128/spectrum.04010-22)
Supplement: Supplemental file 1 — Fig. S1 to S4. Download spectrum.04010-22-s0001.pdf, PDF file, 1.7 MB [file spectrum.04010-22-s0001.pdf]

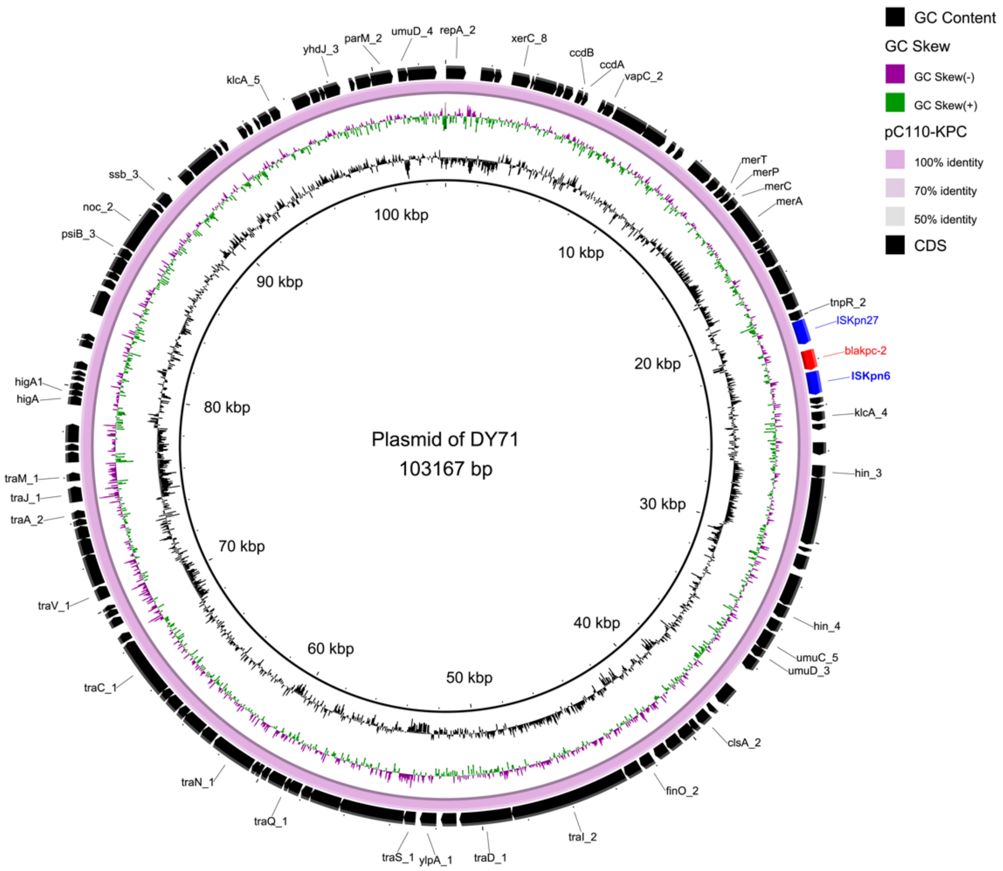

Figure S1: Comparison of *bla*$_{KPC-2}$-carrying plasmid of DY71with plasmid pC110-KPC (accession number: CP047692.1).

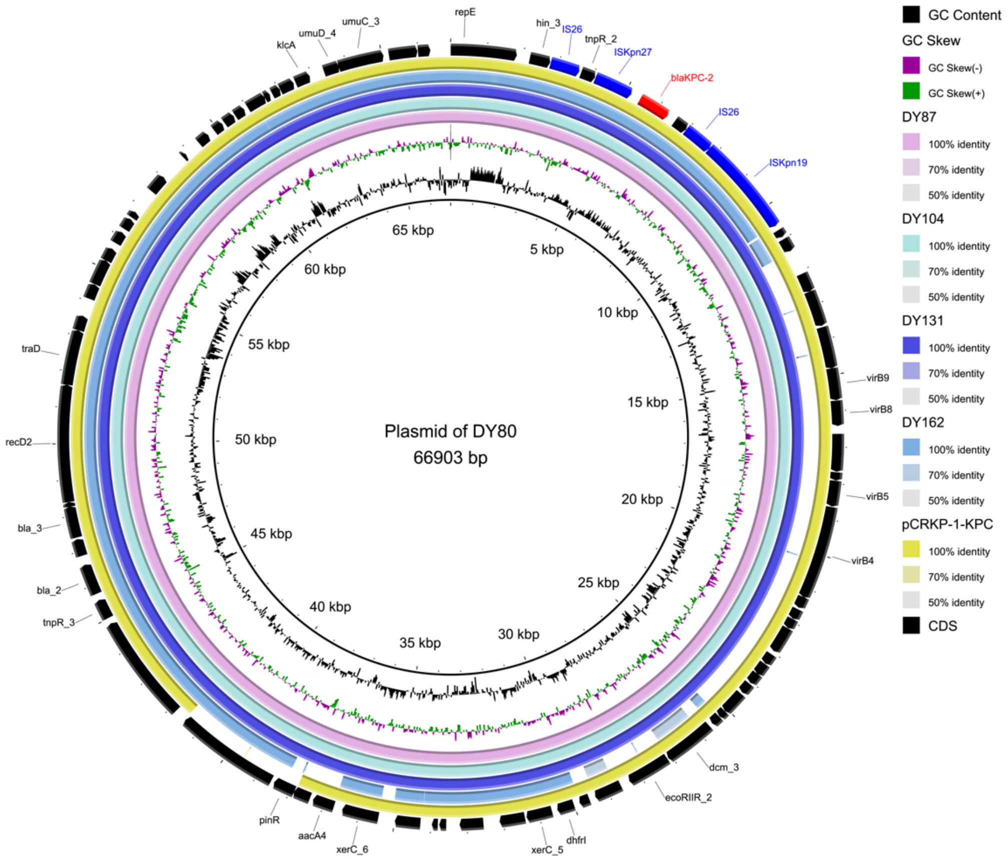

Figure S2: Comparison of *bla*$_{KPC-2}$-carrying plasmid of DY80， DY87, DY104, DY131 and DY162 with plasmid pCRKP-1-KPC (accession number: KX928750.1).

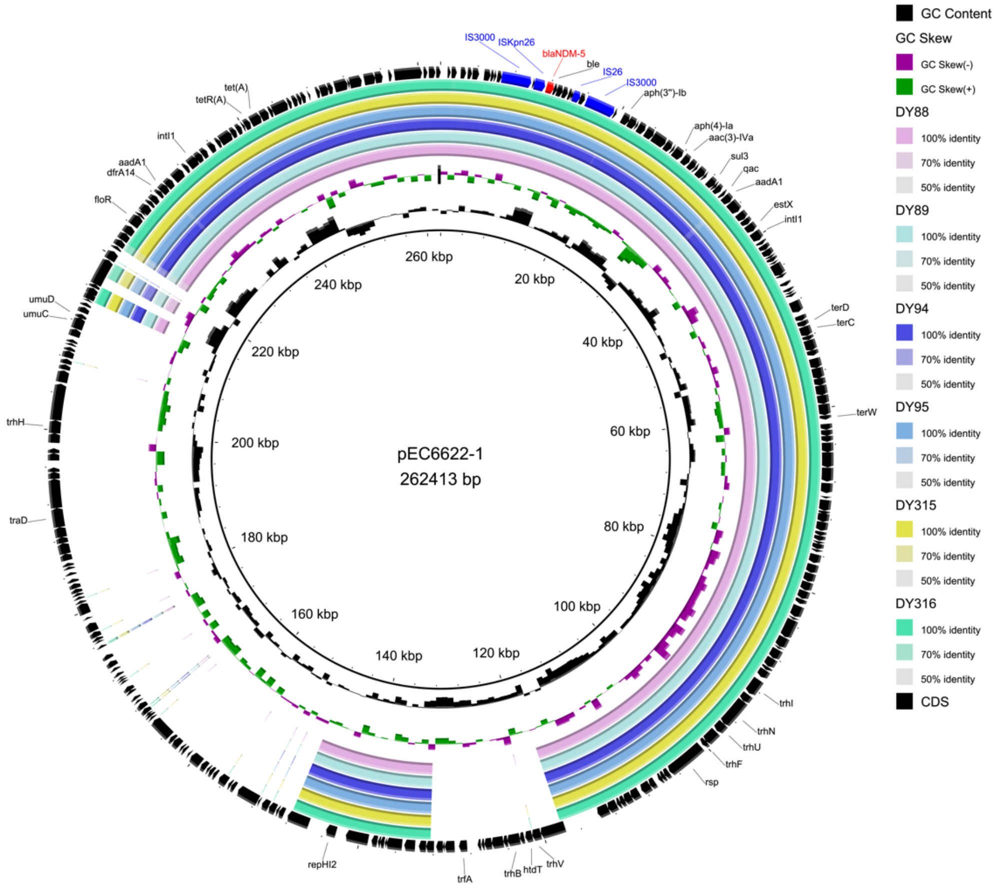

Figure S3: Comparison of *bla*$_{NDM-5}$-carrying plasmid of DY88, DY89, DY94, DY95, DY315 and DY316 with plasmid pEC6622-1 (accession number: CP096588).

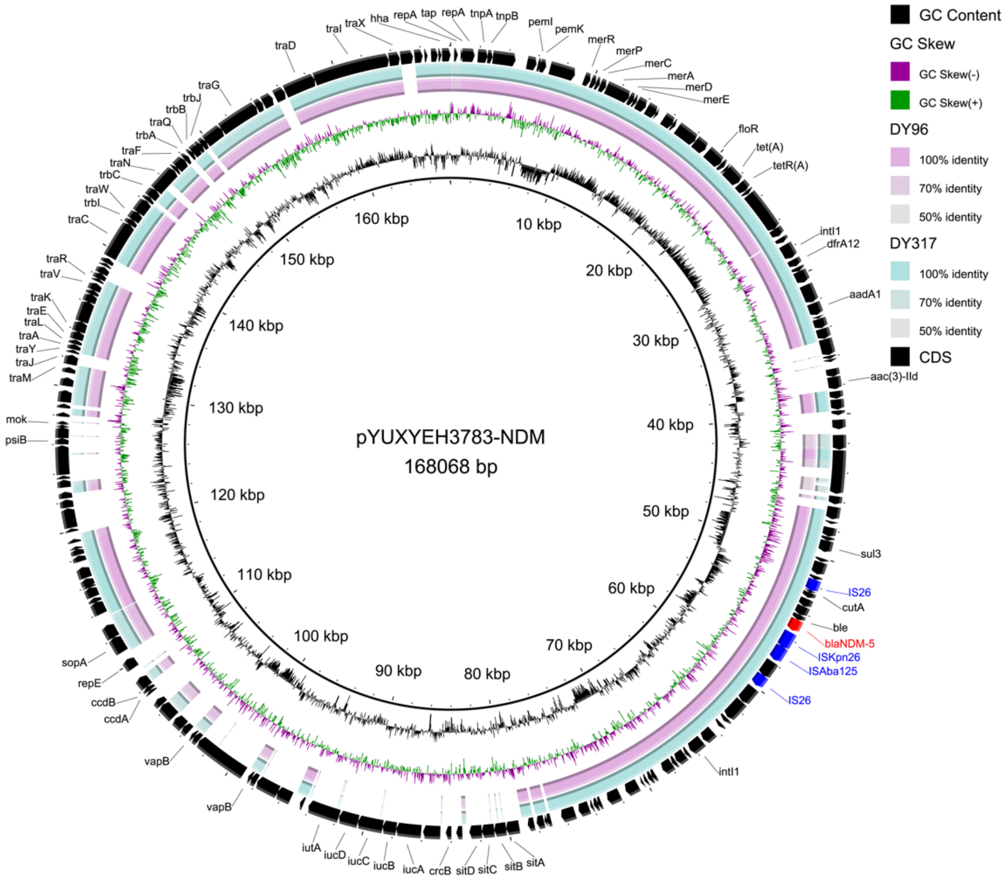

Figure S4: Comparison of *bla*$_{\text{NDM-5}}$-carrying plasmid of DY96 and DY317 with plasmid pYUXYEH3783-NDM (accession number: CP110998).
